# Patient Perception and Cost-Effectiveness of a Patient Navigation Program to Improve Breast Cancer Screening for Hispanic Women

**DOI:** 10.1089/heq.2018.0089

**Published:** 2019-06-20

**Authors:** Yan Li, Erin Carlson, Denise A. Hernández, Brandie Green, Tania Calle, Talitha Kumaresan, Kumbirai Madondo, Mariluz Martinez, Roberto Villarreal, Leah Meraz, José A. Pagán

**Affiliations:** ^1^ Center for Health Innovation, The New York Academy of Medicine, New York, New York.; ^2^ Department of Population Health Science and Policy, Icahn School of Medicine at Mount Sinai, New York, New York.; ^3^ College of Nursing and Health Innovation, The University of Texas at Arlington, Arlington, Texas.; ^4^ College of Architecture, Planning and Public Affairs, The University of Texas at Arlington, Arlington, Texas.; ^5^ Williams College, Williamstown, Massachusetts.; ^6^ Union College, Schenectady, New York.; ^7^ Research and Information Management, University Health System, San Antonio, Texas.; ^8^ Department of Public Health Policy and Management, College of Global Public Health, New York University, New York, New York.; ^9^ Leonard Davis Institute of Health Economics, University of Pennsylvania, Philadelphia, Pennsylvania.

**Keywords:** patient navigation, breast cancer screening, minority health, cost-effectiveness

## Abstract

**Purpose:** Hispanic women are less likely to be screened for breast cancer than non-Hispanic women, which contributes to the disproportionate prevalence of advanced-stage breast cancer in this population group. Patient navigation may be a promising approach to help women overcome the complexity of accessing multiple health care services related to breast cancer screening and treatment. The goal of this study is to assess patient perception and cost-effectiveness of a multilevel, community-based patient navigation program to improve breast cancer screening among Hispanic women in South Texas.

**Methods:** We used mixed methods—including focus groups of program participants and a microsimulation model of breast cancer—to evaluate the effectiveness and cost-effectiveness of the program on the target population. Program data from 2013 to 2016 were collected and used to conduct the analyses.

**Results:** Focus groups showed that the patient navigation program improved patient knowledge, attitudes, and behaviors regarding breast health and increased the mammography screening rate from 60% to 80%. Cost-effectiveness analysis showed that the program could increase life expectancy by 0.71 years and yield an incremental cost-effectiveness ratio of $3120 per quality-adjusted life year compared to no intervention.

**Conclusion:** The 3-year multilevel, community-based patient navigation program effectively increased mammography screening uptake and adherence and improved knowledge and behaviors on breast health among program participants. Future research is needed to translate and disseminate the program to other socioeconomic and demographic groups to test its robustness and design.

## Introduction

While the incidence rate has remained constant and mortality has decreased over the past two decades, breast cancer remains the most common cancer among women.^1^ In the United States, about one in eight women will develop an invasive form of breast cancer, and about 40,610 women are expected to die from breast cancer in 2017.^1^ There are significant disparities in breast cancer screening, incidence, and mortality by socioeconomic status, geography, race, and ethnicity.^2,3^ Although the breast cancer incidence rate for Hispanic women is 40% lower than for non-Hispanic white women, Hispanic women are more likely to be diagnosed at an advanced stage of breast cancer; they are also about 20% more likely to die from it.^4^

Disparities in health outcomes may be explained by differences in mammography utilization and follow-up.^4^ Mammography rates for U.S. women aged 50 to 74 increased dramatically from 36.63% in 1987 to 75.44% in 1998 and have remained at this level over the past two decades.^5^ However, Hispanic women are less likely to be screened for breast cancer than non-Hispanic women,^6,7^ which may contribute to the disproportionate prevalence of advanced-stage disease in this population group.^8^ Reasons for a low breast cancer screening rate among Hispanic women include a lack of knowledge about cancer risk factors and cancer fatalism.^9–11^ Identifying new strategies to increase mammography rates beyond the current plateau and close persisting racial and ethnic screening gaps remains a priority in decreasing breast cancer mortality and health disparities.

Patient navigation may be a promising approach to help women overcome the complexity of accessing multiple health care services related to breast cancer screening and treatment.^12,13^ Communication, insurance, transportation, fear of embarrassment, and concerns about provider cultural sensitivity are all barriers to mammography use among Hispanic women.^12^ Patient navigation programs offer assistance to “underserved populations in ‘navigating’ through the complex health care system to overcome barriers in accessing quality care and treatment” and show promise as a way of increasing mammography use in vulnerable populations.^13^ A variety of services fall under the domain of patient navigation such as assisting with insurance issues, scheduling appointments, coordinating transportation, providing language interpretation, and explaining health information. Patient navigation has been shown to be effective in alleviating anxiety associated with the screening process and, thus, improving screening uptake and adherence.^14^ Recent research also has shown that patient navigation can be strengthened by integrating principles of behavioral economics—such as accurately estimating risk, personalizing information, invoking social norms, and providing incentives—to improve cancer screening in underserved populations.^15,16^

This study assesses patient perception and cost-effectiveness of a patient navigation program implemented in San Antonio, Texas. The program used patient navigators to provide comprehensive breast cancer screening services targeting underserved Hispanic women. Although the program has demonstrated an initial success by increasing the breast cancer screening rate among the target population, it is unknown how patients perceived the program and whether the program can be sustained in the long term with a favorable cost-effectiveness ratio.

To answer these questions, we developed an innovative mixed method approach that combined qualitative focus groups with economic microsimulation. Focus groups would allow us to assess the impact of the program on patient knowledge, attitudes, and behaviors regarding breast health, while a microsimulation model of breast cancer progression would help us determine the long-term health impact and cost-effectiveness of the program. This innovative study design provides a comprehensive evaluation of both the effectiveness and long-term cost-effectiveness of the program, which would be more helpful to practitioners and decision-makers who are interested in implementing a similar program.

## Methods

### Program description

From 2013 to 2016, University Health System (UHS)—the Bexar County Hospital District—implemented the *A Su Salud* Breast Health Program to increase the breast cancer screening rate of Hispanic women. The program primarily targeted Hispanic women 40 years of age and older enrolled in CareLink (a financial assistance program for the uninsured) who have never been screened for breast cancer or have not been screened in the last 5 years. The program included a health promotion media campaign, educational outreach, a patient navigation initiative, and the provision of mammography screening services. The media campaign and educational outreach activities aimed to modify behavior through mass-media education, disseminating breast cancer prevention messages often reinforced by peer role models, as well as by employing an outreach coordinator to educate small businesses, churches, and community groups in the targeted zip codes of the program. Simultaneously, the patient navigation and mammography service components intended to remove social, cultural, and economic barriers by supporting patients through the screening system and providing free services for eligible women.

Approximately 2100 women age 40 and older from our target populations were navigated through the program. In addition, the mammography screening rate for the target population increased from 60% to 80% throughout the program.

### Participant focus groups

Focus groups assessed the knowledge, attitudes, and behaviors of participants, as well as their overall experience, utilizing the breast cancer services from the *A Su Salud* program. Individuals recruited to participate in the focus groups included any individual that received or was currently receiving services through the UHS *A Su Salud* Breast Health Program. Additional eligibility criteria included participants fluent in English or Spanish and willing to speak openly in a group setting about their experience with the program. Patient navigators contacted individuals who participated in the program during any of the three program years through telephone to recruit them to participate in the focus groups. All eligible individuals received mailed postcard invitations to participate in the focus groups. If interested, individuals called the phone number provided on the post card to connect with a navigator. Individuals who agreed to participate received information from patient navigators about the location and time for their focus group, assigned according to the participant's preferred language. All focus group participants received a gift card as compensation for their time and participation.

Five focus groups consisting of 30 total participants were conducted in April 2016. Two focus groups were conducted in English, and three focus groups were conducted in Spanish. Each focus group was recorded and transcribed. Using an iterative method of content analysis utilized frequently in qualitative research, researchers coded transcripts for themes using NVivo 11 software. Before coding, each reviewer first read the transcripts in their entirety to allow for ideas and concepts to develop. Each of the two reviewers identified emerging themes individually and met to discuss initial findings. After initial discussions, reviewers went through transcripts to identify specific subthemes. After several meetings, reviewers finalized the code tree and codebook utilized for coding the focus group transcripts. The code tree and codebook served as a tool to allow for better agreement between coders. Throughout the coding process, coders communicated to discuss challenges, emerging themes, and agree on conversations that were difficult to code. Intercoder reliability found 0.78 percent agreement among coders. The project received Institutional Review Board approval from University of North Texas Health Science Center.

### Cost-effectiveness analysis

We used a stochastic microsimulation model to evaluate the long-term health and economic consequences associated with the *A Su Salud* Breast Health Program. We modeled the natural history of breast cancer based on a synthesis of evidence from existing breast cancer simulation models. In particular, we developed the main model structure and estimated disease progression transitions based on a model developed by researchers from the University of Minnesota and the University of California, San Francisco.^17,18^ Data on program costs, population characteristics (e.g., age distribution), and screening rates before and after the program implementation were collected from the breast health program. Other model parameters (e.g., quality-of-life utilities) were obtained from other existing literature.^19–22^ We also reviewed the Cancer Intervention and Surveillance Network (CISNET) breast cancer models to ensure that our model structure and parameter estimation reflect the state of the art in breast cancer disease progression modeling.^23–25^

The microsimulation model captured the potential breast cancer disease progression for a simulated population cohort and calculated discounted costs and quality-adjusted life years (QALYs) throughout their lifetime. We simulated a large population cohort (i.e., 100,000 individuals) for two scenarios modeled (i.e., the *A Su Salud* program and status quo), which would help reduce stochastic uncertainties and provide us with stable estimates of long-term outcomes. We reported lifetime costs and QALYs and discounted them by 3% annually. A discount rate of 3% is commonly used in cost-effectiveness analysis in health care and medicine.^26^ We also reported life expectancy and the incremental cost-effectiveness ratio (ICER). An ICER measures the additional cost resulting from the breast health program that must be incurred to gain an additional QALY relative to the status quo. As a common standard in the United States, we used $50,000 per QALY as a threshold to determine cost-effectiveness of the studied program.^27^ In other words, a program is considered cost-effective if it has an ICER less than $50,000 per QALY compared to the status quo. Detailed model documentation is available upon request.

## Results

### Patient focus groups

Table 1 reports demographic and socioeconomic information of the 30 focus group participants. Three out of every five participants were born outside the United States (61% of them had been in the United States for 20 years or less). Thirty percent of focus group participants had less than a high school diploma. More than half of focus group participants (56%) earned less than $1000 per month; almost half of them (45%) were unemployed or out of the labor force. Seventy percent of participants had or knew someone diagnosed with cancer at some point in their lives. Half of focus group participants reported having fair or poor health.

**Table 1.** Characteristics of the Focus Group Participants

**Characteristic**	**No. of participants (*n*=30)**	**Percent of total**
Born outside the United States	18	60
Education level		
Less than high school	9	30
High school or technical training	11	37
College of higher	10	33
Monthly income		
Less than $1000	17	56
$1000–$1500	8	27
More than $1500	5	17
Employment		
Employed	14	46
Unemployed	14	46
Did not answer	2	8
Have a primary care doctor?		
Yes	27	90
Had or knew anyone diagnosed with cancer?		
Yes	21	70
Had or knew someone diagnosed with breast cancer?		
Yes	16	53
Self-reported health		
Excellent	3	10
Good	12	40
Fair	13	43
Poor	2	7

#### Knowledge related to breast health

Many focus group participants stated that they obtained information about breast health from a health care professional or from the Internet. Participants also discussed their depth of knowledge regarding breast cancer screening, including mammograms and breast self-examinations. Although most of the participants indicated that they had the knowledge to perform a self-examination or knew where to obtain an examination, many of them requested additional information related to the frequency of screening, as well as the age to initiate mammograms:

I had some questions and I was given information on how to look up certain things. I had problems with pain and sensitivity. So, I was given pamphlets and websites to go and look up and talk to my doctor also about what was going on. (Participant)

Risk factors for breast cancer were also discussed by participants within each focus group, with heredity being the most-often mentioned risk factor for breast cancer. How breast cancer develops was another topic that was a discussion point during the focus groups. Some of the participants indicated that breast cancer develops in the breast tissue and by cancer cells that are in the body. Some of the other participants were unsure and requested additional information.

#### Attitudes toward breast health

Attitudes regarding breast health were assessed according to whether or not participants favor or disfavor mammograms and breast self-examinations, as well as views on susceptibility for developing breast cancer. Overall, most of the participants had a positive attitude toward both mammograms and breast self-examinations. Many of the participants who desired screening indicated that they were more susceptible to developing breast cancer due to their family history, preexisting conditions, ethnicity, lifestyle, or current health status. Those participants relating a distaste toward screenings indicated that cultural beliefs, fear of associated pain, false negatives, and apprehension led them to avoid screenings.

I think that the positive is that we're avoiding getting sick from cancer if we find it in time. The negative is a lot of people don't do it and when they do it it's already late and they don't have a cure and it's when the person dies. So to avoid that I say we have to be checking ourselves, go to the doctor, do our exams every day not only or say yes I'm going to do it once a week because you never know. Everybody is different, every person is different. I think that the most important is to get checked to avoid the negative that it's too late. That's what I say. (Participant)

Participants also offered insight on how elements of their personal life such as cultural and generational norms, family practices, experiences, and preferences influence their assessment of breast health services and screening behaviors. Of all the factors considered, culture was repeatedly mentioned as one important determinant of breast health knowledge, breast health screening behavior, and the likelihood of developing cancer. Focus group participants believed that there is a lack of information within their own culture that may be due to embarrassment regarding the topic, which results in the lack of discussion about breast health. Participants also reported believing that Hispanic women were not as concerned about taking care of themselves or visiting the doctor. Overall, participants believed that minority women, specifically Hispanic, African American, and Muslim women, were more susceptible to developing breast cancer than their White counterparts:

I believe there is a racial significance that we get breast cancer, the darker the skin, the more susceptible we are, at least that's what I think I read. That racial barrier or racial consequences do play a part in getting breast cancer. (Participant)

#### Health behaviors

Participants offered their views on their individual health behavior practices, including information sharing, need for lifestyle changes, and intention to continue obtaining breast cancer screening. Most of the participants agreed that they discuss any information regarding their breast health with their health care provider. Some focus group participants also anticipated making a lifestyle change as a result of program participation. Lifestyle changes mentioned included losing weight, modifying diet, becoming more active, and quitting smoking.

It's not just the diet. It's lifestyle changes. When I was saying exercising, I wasn't talking about food, I was talking about lifestyle choices. How much do you drink? How much do you smoke? Or do you have to do those things? Those are some of the things that we can modify and that's what the screenings are about. It's not just, “we are going to check your breasts.” These are the things that will put you in a risk factor, for not just cancer of the breast, but other problems. (Participant)

### Cost-effectiveness analysis

Table 2 presents the results from the cost-effectiveness analysis of the *A Su Salud* Breast Health Program. The *A Su Salud* Breast Health Program would cost an average of $124.80 more than the status quo per person over the lifetime of participants. The program would increase the life expectancy of the population cohort by 0.71 years and increase QALYs by 0.04 years. The ICER for the breast health program relative to the status quo was $3120 per QALY. Since $3120 per QALY is less than the $50,000 per QALY threshold, we conclude that the breast health program is highly cost-effective under the parameter assumptions described above.

**Table 2.** Cost-Effectiveness of *A Su Salud* Breast Health Program Versus Status Quo

	**Cost ($)**	**Life expectancy**	**QALY**	**ICER ($/QALY)**
Breast health program	2632.90	23.03	14.09	3120.00
Status quo	2508.10	22.32	14.05	
Incremental cost or effectiveness	124.80	0.71	0.04	

ICER, incremental cost-effectiveness ratio; QALY, quality-adjusted life year.

**FIG. 1.**
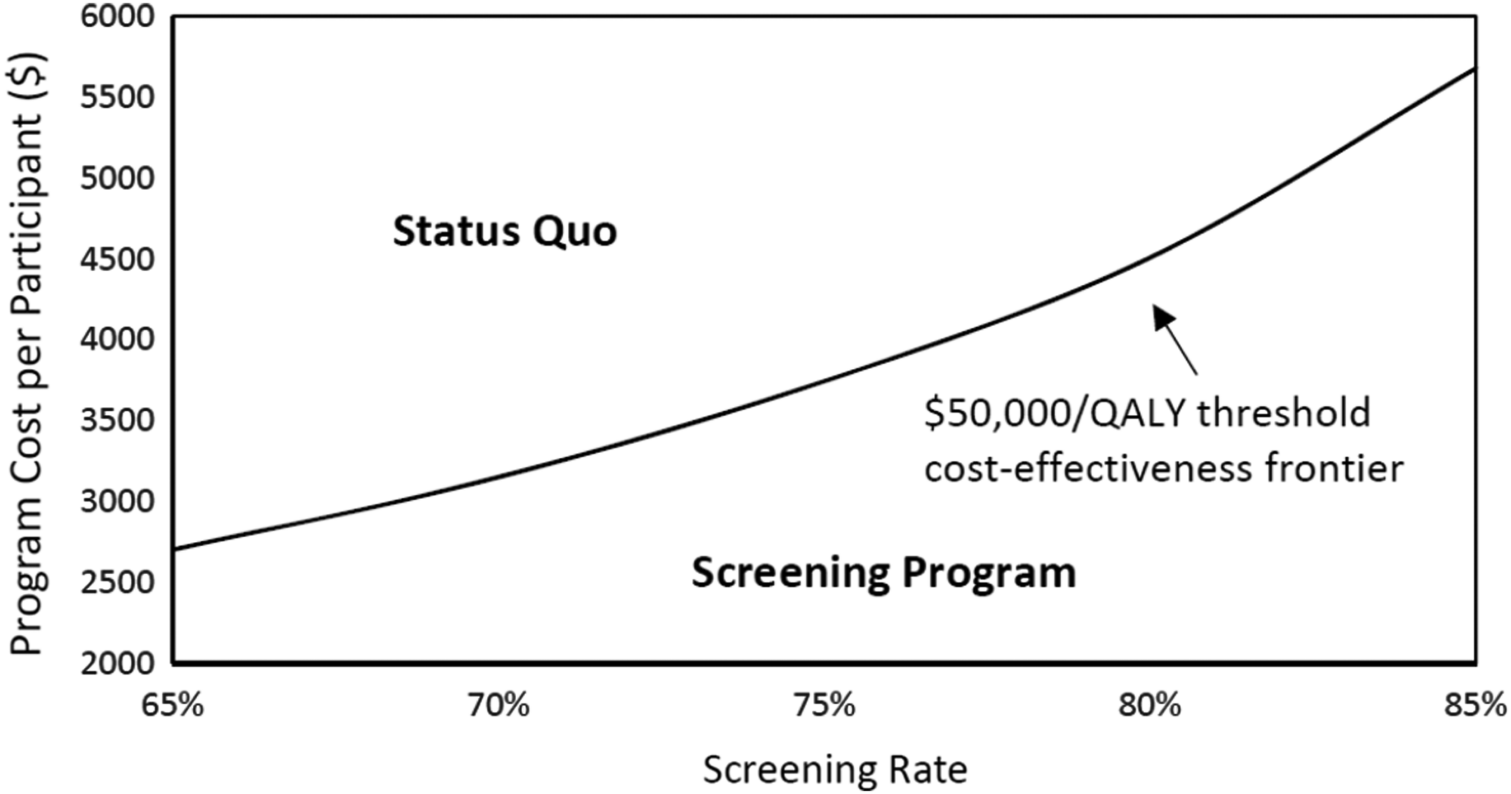
Two-way sensitivity analysis of the program cost per participant and screening rate for the choice of status quo or screening program given the $50,000/QALY cost-effectiveness threshold. QALY, quality-adjusted life year.

We also studied the robustness of the baseline results by conducting sensitivity analyses that account for uncertainties in the costs and effectiveness of the *A Su Salud* Breast Health Program. Figure 1 reports the results of a two-way sensitivity analysis of the breast health program cost per participant and the screening rate. If a combination of program cost per participant and screening rate falls below the cost-effectiveness frontier in Figure 1, the ICER of the breast health screening program relative to status quo is less than $50,000 per QALY, which means the program is more cost-effective; otherwise, the status quo is more cost-effective. The results show that as the 3-year breast cancer screening rate increased from 60% to 80%, the cost of the program could increase up to five times—from $774 to $4508—before the program becomes less cost-effective. In addition, even if the breast health program only results in a screening rate of 65% (a 5% increase from the status quo), the program will be cost-effective as long as its cost remains less than $2702 per person.

## Discussion

The findings from our study suggest that the *A Su Salud* Breast Health Program and programs like it may be able to increase the mammography screening rate and life expectancy of Hispanic women and other vulnerable populations by improving patient knowledge, attitudes, and behaviors regarding breast health. Across focus group responses, participants indicated that they improved their knowledge of risk factors, available resources, and general health practices, which are important for breast cancer screening adherence. Participants also acknowledged that many impediments to care—such as fear of procedure(s), false negatives, and cultural beliefs—were lessened as a result of program participation. Participant behaviors also improved; of those who responded to a question related to their intention to maintain breast health screenings, all replied affirmatively. These findings provide a comprehensive picture of the reasons why the screening rate was increased significantly due to the breast health program.

Results from the cost-effectiveness analysis further showed that the program was effective in increasing the life expectancy and quality of life for program participants in a cost-effective manner. Specifically, the breast health program could increase the life expectancy of each participant by 0.71 years while the cost of the program could increase up to five times before the program is no longer cost-effective.

Similar multilevel, community-based patient navigation programs have been shown to improve the cervical cancer screening rate of Hispanic women and the colorectal cancer screening rate of Hispanic men.^28,29^ In these previous studies, we showed that patient navigation programs targeting cervical cancer and colorectal cancer were also cost-effective due to significantly reduced cancer risk.^28,29^

These programs share several elements in common: they were culturally sensitive and comprehensive,^28,29^ providing the underserved Hispanic population with more knowledge about cancer risk factors and reducing the prevalence of cancer fatalism within this population.^9,11^ They also used principles from behavioral economics—such as providing personalized risk assessment to address *unrealistic optimism* of some patients and reminding patients of their missed appointments (*allowance for errors*)—to engage participants and increase cancer screening uptake and adherence.^16,28,29^ Behavioral economics, which emphasizes the effects of psychological, social, and cognitive factors on the decisions of individuals, has demonstrated great promise in improving cancer screening for underserved populations.^15^ Based on these promising results, health care providers may consider developing and testing similar programs in other places or for other populations of interest to improve cancer screening uptake and reduce disparities among underserved populations.

There are a number of limitations in this study. First, interview participant responses may be intentionally or unintentionally biased. Second, evaluation interviews are not generalizable to the larger population outside of UHS because random sampling methods were not used to select participants. Third, the breast cancer natural history model was developed based on parameters that reflect the general U.S. population, not specifically the target population of this study. We have conducted sensitivity analyses to account for the uncertainty in simulation results. We will further improve the results as more local data for model parameterization become available and conduct more comprehensive sensitivity analyses.

In conclusion, this study has demonstrated the effectiveness and cost-effectiveness of a multilevel, community-based patient navigation program implemented by a health system serving a low-income Hispanic population. The program successfully integrated a health promotion media campaign, educational outreach, a patient navigation initiative, and the provision of mammography screening services. Our positive findings indicate that the patient navigation program could be translated to other socioeconomic and demographic groups. Future work needs to be done to better understand which of the behavioral change components in the program are most promising.

## Abbreviations Used

CISNET: Cancer Intervention and Surveillance Network
ICER: incremental cost-effectiveness ratio
QALY: quality-adjusted life year
UHS: University Health System
